# Subcellular Proteome Analysis Reveals Apoptotic Vulnerability of T-Cell Acute Lymphoblastic Leukemia

**DOI:** 10.1155/2022/5504475

**Published:** 2022-04-15

**Authors:** Xiaolei Song, Xiaojing Wu, Zihan Zhang, Zhangxiu Cui, Yong Zheng, Jian Sun

**Affiliations:** ^1^Department of Molecular and Cellular Pharmacology, School of Pharmaceutical Science and Technology, Tianjin University, Tianjin 300072, China; ^2^State Key Laboratory of Proteomics, Beijing Proteome Research Center, National Center for Protein Sciences (Beijing), Beijing Institute of Lifeomics, Beijing 102206, China

## Abstract

Targeting death receptor-mediated apoptosis in T-cell acute lymphoblastic leukemia (T-ALL), an aggressive disease with poor prognosis, is hindered by the inherent resistance of primary leukemia cells. Knowledge on therapeutic vulnerabilities in these malignant cells will provide opportunities for developing novel combinatory treatments for patients. Using label-free quantitative mass spectrometry and subcellular fractionation techniques, we systematically compared organelle-specific proteomes between Jurkat cells, an *in vitro* model for T-ALL, and a Jurkat mutant with increased resistance to death receptor-mediated apoptosis. By identifying several differentially regulated protein clusters, our data argued that extensive metabolic reprograming in the mitochondria, characterized by enhanced respiration and energy production, might allow cells to evade DR5-mediated cytotoxicity. Further analysis using clinical datasets demonstrated that the elevated expression of a three-gene signature, consisting of SDHA, IDH3A, and ANXA11, was significantly associated with poor survival of acute leukemia patients. Our analysis therefore provided a unique dataset for a mechanistic understanding of T-ALL and for the design of novel ALL treatments.

## 1. Introduction

T-cell acute lymphoblastic leukemia (T-ALL), accounting for ~25% of all acute lymphoblastic leukemia (ALL), is a rare and aggressive disease of the bone marrow. Compared to pediatric T-ALL, the prognosis is usually much worse for elder patients, where it is primarily diagnosed with highly limited treatment options [1–3].

Dysregulated apoptosis is a hallmark for virtually all malignancies, yet specific mechanisms are tumor type-specific [4, 5]. In hematological malignancies such as leukemia, evasion from apoptosis is often achieved by systematic deregulation of two separate but related apoptotic signaling pathways: the intrinsic pathway is mainly controlled by BCL-2 family proteins and converges on the mitochondria [6], while the extrinsic pathway is activated by TNF family receptors such as death receptor 4 (DR4), death receptor 5 (DR5), or FAS upon receiving extracellular cues [7]. The promise of targeting apoptotic pathways as a feasible therapeutic strategy in leukemia has been recently demonstrated by the approval of venetoclax, a BH3-mimetic to inhibit the antiapoptotic molecule Bcl-2. However, no inhibitors within this class have been approved for T-ALL, although preclinical studies using childhood ALL xenografts showed potentials [8]. While the biological role of the extrinsic pathway in ALL remains elusive, death receptor-mediated signaling recently emerged as an attractive target for disease intervention [9]. DR5 (also known as TNFRSF10B) is a type II membrane receptor that has significantly elevated expression in numerous tumors but not normal tissues. Upon ligation by its natural ligand, the tumor necrosis factor-related apoptosis-inducing ligand (TRAIL), DR5 is activated through oligomerization and catalyzes the formation of the death-inducing signaling complex (DISC), which in turn activates downstream signaling events to induce apoptosis in both mitochondrial-dependent and mitochondrial-independent manners. Due to their tumor-specific cytotoxicity, several humanized DR5-agonistic monoclonal antibodies have been developed and approved to be able to induce apoptosis in various tumor models [5–10]. Indeed, a number of DR5 targeting agents, including multivalent antibodies, antibody-drug conjugates, recombinant TRAIL variants, and small molecules are currently under active clinical evaluations with promising results in a number of cancers, demonstrating the therapeutic value of DR5 [11–16]. Notably, the human T-ALL cell lines such as Jurkat are among the most sensitive cells towards in vitro and in vivo cytotoxicity of anti-DR5 antibodies, providing initial support for the feasibility of their applications in T-ALL treatments.

However, clinical trials using DR5-agonistic antibodies, either as monotherapy or combined with chemotherapy or other targeted therapies, have been failed to show benefits in leukemia, although safety profiles have been well documented [17]. A key reason for this disappointment is due to the inherent resistance to DR5-induced apoptosis in primary leukemia cells [18]. Mechanisms mediating the resistance remain elusive, partly due to the lack of systematic studies to identify previously unappreciated signaling nodes critical for proper propagation of death receptor signaling [19]. To systematically address this issue, we first established a mutant Jurkat with drug-induced resistance toward zaptuzumab, a novel DR5-agnoistic humanized monoclonal antibody [15, 20]. Next, we quantitatively investigated proteomic difference between wild-type and mutant Jurkat cells using organelle-specific proteome analysis and label-free quantitative mass spectrometry. The high genetic similarity between these two cell models allowed us to identify several potential proteomic vulnerable points, characterized by enhanced respiration and energy metabolism, in resistant cells to confer resistance for DR5-targeted therapies. Further analysis using clinical data from TCGA database identified a three-gene signature, consisting of proteins involved in energy metabolism, as a novel prognosis marker for acute leukemia. Our work provided a rich referential dataset for the design of novel DR5-targeting therapies for T-ALL.

## 2. Materials and Methods

### 2.1. Materials and Reagents

Cell Counting Kit-8 was from Med ChemExpress (NJ, USA). Minute™ Cell Fractionation and Protein Trafficking kit was from Invent(MN, USA). Formic acid (FA) was from Solarbio (Beijing, China). Protease and phosphatase inhibitor cocktails were from Roche (Basel, Switzerland). Trifluoroacetic acid (TFA), trypsin, and chemical inhibitors were from Sigma (St. Louis, MO, USA). Water was purified using a Milli-Q system from Millipore Co. (Bedford, MA).

### 2.2. Cell Culture and Drug Treatment

Jurkat cells from ATCC were grown in RPMI 1640 medium, supplemented with 10% fetal bovine serum, 100 U/mL of streptomycin, and 100 U/mL of penicillin in a humid atmosphere (5% CO_2_ at 37°C). To induce drug resistance, wild-type cells were sequentially challenged with growth medium supplemented with increasing amounts of zaptuzumab (a kind gift from D. Zheng, PUMC, China) with an initial dose of 1.5 ng/mL and one week interval. After a ten-round subculturing, the resulting zaptuzumab-resistant variant, named as JurkatR, could proliferate in 15000 ng/mL zaptuzumab.

### 2.3. Cell Viability Determination

The cells were seeded at 1 × 105/100*μ*L in a 96-well plate and incubated overnight at 37°C. After exposed to the indicated concentrations of zaptuzumab for two hours, cell viabilities were measured using Cell Counting Kit-8, following the manufacturer's instructions.

### 2.4. Subcellular Organelle Enrichment by Differential Centrifugation

Subcellular fractions were obtained using the Minute™ Cell Fractionation and Protein Trafficking kit. 5 × 107/sample Jurkat cells were used. Cell lysis and subsequent preparation of subcellular fractions based on differential centrifugation were following the manufacturer's instructions.

### 2.5. Immunoblotting

Samples were mixed with SDS-PAGE sample buffer and boiled. Proteins were separated on a 10% SDS-PAGE and transferred onto a nitrocellulose membrane (Amersham Biosciences) at 100 V for 90 min. Indicated proteins were identified by incubating with specific antibodies and ECL.

### 2.6. Mass Spectrometry Sample Preparation

Subcellular fractions were concentrated using 10 kDa ultrafiltration, resuspended in 50 mM ABC, and tryptic digested. After 14000 g centrifugation, the filtrates were desalted using a “spin-tip”-modified solid phase with C18 filter membrane and T4 pipette tip, followed by washing with 50% ACN/0.1% TFA and 0.1% TFA, respectively, before eluting with 70% ACN and lyophilization.

### 2.7. Mass Spectrometric Analysis and Database Search

Samples were reconstituted with 3% formic acid and automatically loaded onto the C18 pulled column (ID at 75 *μ*m) packed with 3 mm ReproSil C18, separated at a flow rate of 200 nL/min and a gradient of 2% to 35% acetonitrile over 90 min, and analyzed by mass spectrometry (TripleTOF 5600, AB SCIEX). Acquired raw files were analyzed by MaxQuant software (version MaxQuant 1.6.2.0) using Andromeda search engine and matched with UniProt human database (version human_protein_faa_2013_0704.fasta) for identification and label-free quantification.

### 2.8. Data Processing

For Gene Ontology analysis, website tool of David (website: https://david.ncifcrf.gov/tools.jsp) was used. The Kaplan-Meier survival analysis and mRNA expression comparison in clinical samples were performed using GEPIA2 (website: http://gepia2.cancer-pku.cn), an interactive web server for analyzing the RNA sequencing expression data of tumor and normal samples from The Cancer Genome Atlas (TCGA) and the Genotype-Tissue Expression (GTEx) projects [21]. Protein expression comparison in tumors was performed with UALCAN (website: http://ualcan.path.uab.edu), an web-based server for analyzing mass spectrometry data of tumor and nontumor tissue samples from the Clinical Proteomic Tumor Analysis Consortium (CPTAC) project [22].

## 3. Results

### 3.1. Inducing Resistance to DR5-Dependent Cytotoxicity in Jurkat Cells

**Figure 1:**
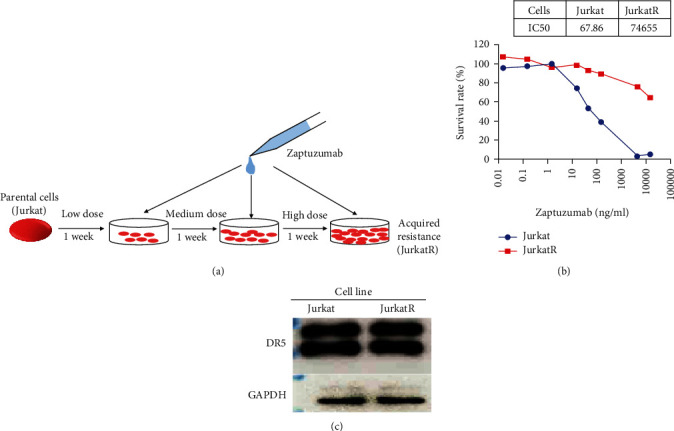
The isolation of a Jurkat-derived mutant resistant to DR5-agonistic antibody-mediated cytotoxicity. (a) Workflow of establishing Jurkat mutant (JurkatR) with decreased sensitivity to zaptuzumab, a DR5-specific agonistic antibody. (b) Sensitivity of wild-type Jurkat and JurkatR to zaptuzumab was determined using the CCK8 method. IC50 values were indicated.

The human acute lymphoblastic leukemia cell line Jurkat is used as the *in vitro* model for T-ALL in our study. This cell line is highly sensitive to the cytotoxic effect of a DR5-agonistic monoclonal antibody named zaptuzumab. To establish a Jurkat variant resistant to zaptuzumab-mediated killing, wild-type cells were subjected to long-term subculturing with increasing doses of zaptuzumab, to allow the spontaneous development of acquired drug resistance (Figures 1(a) and 1(b)). This results in the isolation of a Jurkat-derived mutant cell line (termed JurkatR thereafter) that has a significantly increased IC50 towards zaptuzumab. The altered drug resistance was not likely caused by DR5 receptor downregulation as the mutant cell maintained a similar level of receptor expression as the wild-type counterpart (Figure 1(c)). The genetic similarity between parental Jurkat and its mutants facilitates the identification of proteomic alterations associated with drug resistance.

### 3.2. Organelle-Specific Proteome Mapping in Wild-Type and Mutant Cells

**Figure 2:**
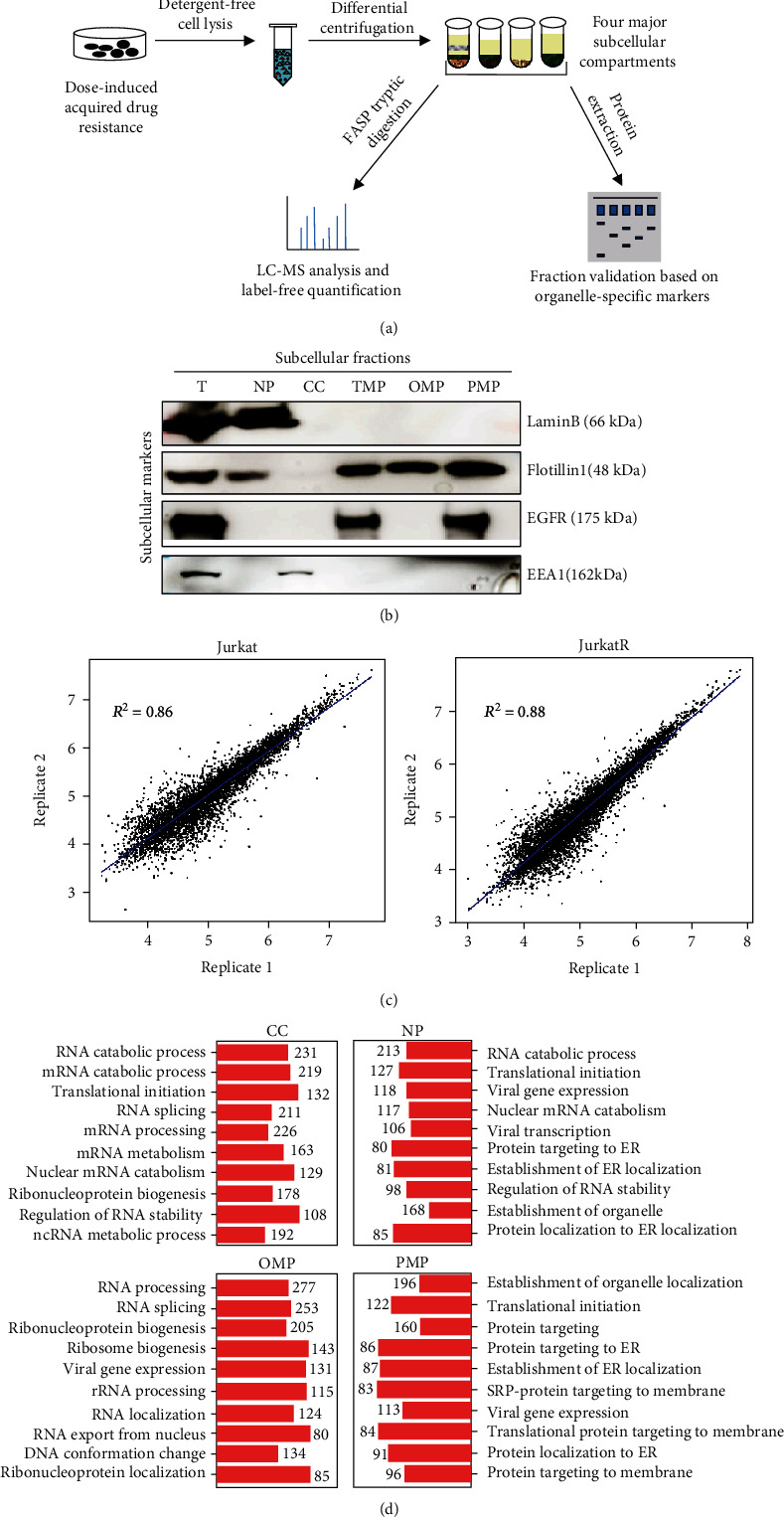
Subcellular organelle-specific proteome analysis. (a) The workflow. (b) Subcellular fractions from Jurkat cells were prepared (see Materials and Methods for details) and validated using specific antibodies against indicated organelle markers by immunoblotting. (c) Linear regression analysis of two independent biological replicates. Pearson's correlation coefficients (*R*^2^) were shown. (d) GO results on MS analysis of four subcellular fractions prepared from JurkatR cells. The results are representative of two independent biological replicates.

Similar to other types of membrane receptors, activated death receptors transduce extracellular signals to eventually activate nuclear gene transcriptions via a sophisticated intracellular signaling network which is regulated at multiple subcellular compartments. To have an in-depth coverage of the subcellular localization-specific proteome, Jurkat and JurkatR cells were resuspended in detergent-free hypotonic buffer and lysed by following the manual of the Minute subcellular organelle separation kit (Invent Biotech), a patented technique for efficient cell lysis by mild physical force to minimize unwanted lysis of organelles. Differential centrifugations were used to recover five subcellular fractions enriched with plasma membrane protein (referred as PMP), organelle membrane protein (referred as OMP), cytosolic protein (referred as CC), and nuclear protein (referred as NP) (Figure 2(a)). The purity of each subcellular fraction was evaluated by distributions of representative organelle-specific markers using specific antibodies and immunoblotting (Figure 2(b)). The results confirmed a sufficient separation between fractions. Next, total proteins from each fraction were extracted, tryptic digested according to the FASP protocol, and analyzed in one run by LC-MS (5600, AB SCIEX). Raw data files were subsequently analyzed by the MaxQuant software using the Andromeda search engine to determine protein identities as well as their relative abundance by label-free quantification (see Materials and Methods for details). The results from two independent biological replicates were highly reproducible, as examined by the average Pearson correlation coefficients (*R*^2^ = 0.86 for Jurkat; *R*^2^ = 0.88 for JurkatR) (Figure 2(c)). To systematically evaluate the quality of prepared subcellular fractions, Gene Ontology (GO) enrichment analysis was performed on identified proteins from each subcellular fraction. As demonstrated by the biological process annotation for most enriched subsets of proteins, the molecular profiles were apparently fraction specific, validating again our fraction preparations (Figure 2(d) and Fig. S1).

### 3.3. Systematic Signaling Network Rewiring and Enhanced Mitochondrial Functions in Cells Resistant to DR5-Mediated Apoptosis

**Figure 3:**
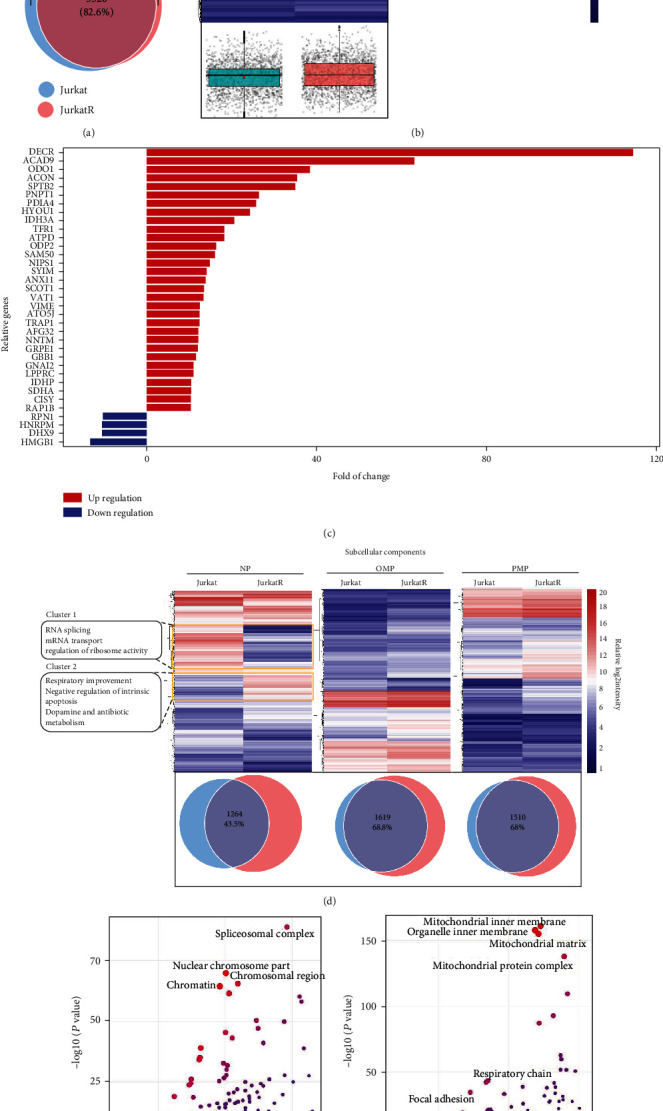
Enhanced respiration and energy metabolism in cells resistant to DR5-induced apoptosis. (a) Venn diagram analysis on total number of overlapped proteins identified in Jurkat and JurkatR after pooling data from four subcellular fractions. (b) Upper part: unsupervised hierarchical clustering of all quantified proteins by label-free MS from Jurkat and JurkatR. Lower part: the average intensities distribution of identified proteins from two cell lines. Data were log2 transformed and significance was assessed using Mann-Whitney *U* (unpaired and nonparametric) test. (c) Most significantly regulated proteins in JurkatR. The *x*-axis: ratio of protein intensity in JurkatR over the corresponding protein intensity in Jurkat. Red: ratio > 10 (upregulated); blue: ratio < 0.1 (downregulated). (d) Upper part: unsupervised hierarchical clustering of proteins identified in indicated subcellular fractions from Jurkat and JurkatR. Lower part: Venn diagram showed the overlaps of proteins identified in the corresponding subcellular fractions. (e) GO analysis (cellular component) on proteins identified in NP fractions from Jurkat and JurkatR. The *x*-axis: enrichment ratios were log2 transformed. The *y*-axis: enrichment –log10 of significant *p* value.

To identify molecular characteristics that may associate with altered drug sensitivity in mutated Jurkat cells, we compared the size of their proteomes by Venn diagram analysis. After combining MS data searching files from four subcellular fractions, a total of 4,098 and 3,685 unique proteins were identified in Jurkat and JurkatR, respectively (peptide and protein FDR at 1%). Overall, the two proteomes have a 82.6% overlap (Figure 3(a)), considerably higher than those observed in genetically unrelated cells, where over two-thirds of the proteomes are different [23]. While the data is consistent with JurkatR being a Jurkat derivative, it is nevertheless surprising that as high as 17.4% of the proteome need to be changed for the cell to evade DR5-induced apoptosis, emphasizing the need for a systematic approach for understanding mechanisms of drug resistance. Moreover, the two proteomes also exhibited apparent quantitative differences, as judged by the dynamic range of protein abundance (Figure 3(b), lower panel). A further unsupervised hierarchical clustering analysis using label-free quantification data identified three significantly altered protein clusters in JurkatR cells (Figure 3(b), upper panel). GO enrichment analysis indicated that proteins consisting the clusters 1 and 3, significantly decreased in JurkatR, mainly involved in regulating gene transcription and p53 signaling, whereas most proteins in cluster 2, upregulated in mutated cells, belonged to the respiration and energy metabolism machinery. This observation is further supported by the analysis of most up- or downregulated proteins (folds of change > 3; Figure 3(c) and Fig. S3). Interestingly, when organelle-specific proteomes between two cells were compared separately, the overlap in number of identified proteins decreased to between 43.5 and 68.8%, where the NP-specific proteome being the most variable and the PMP and the OMP-specific proteomes being the most stable between cells (Figure 3(d), lower panel, and Fig. S2). This observation was further supported by unsupervised hierarchical clustering analysis on the NP-specific proteome (the most variable) and the OMP/PMM (the least variable proteome) (Figure 3(d), upper panel). These results strongly argued that the altered spatial organization of subcellular proteomes in JurkatR played a more important role in shaping cellular functions. We then performed GO analysis on the most changed NP-specific proteome and identified two protein clusters similarly reflecting protein patterns previously described in Figure 3(b), where proteins involved in gene transcription were downregulated while those involved in regulating the respiration chain and energy metabolism (Figure 3(d)), mostly found in the mitochondria (Figure 3(e)), are upregulated.

Taken together, the above analysis identified that enhanced mitochondrial function might serve as a key mechanism for cells to acquire resistance for DR5-related apoptosis.

### 3.4. Identification of a Three-Gene Signature as a Prognosis Marker for Acute Leukemia

**Figure 4:**
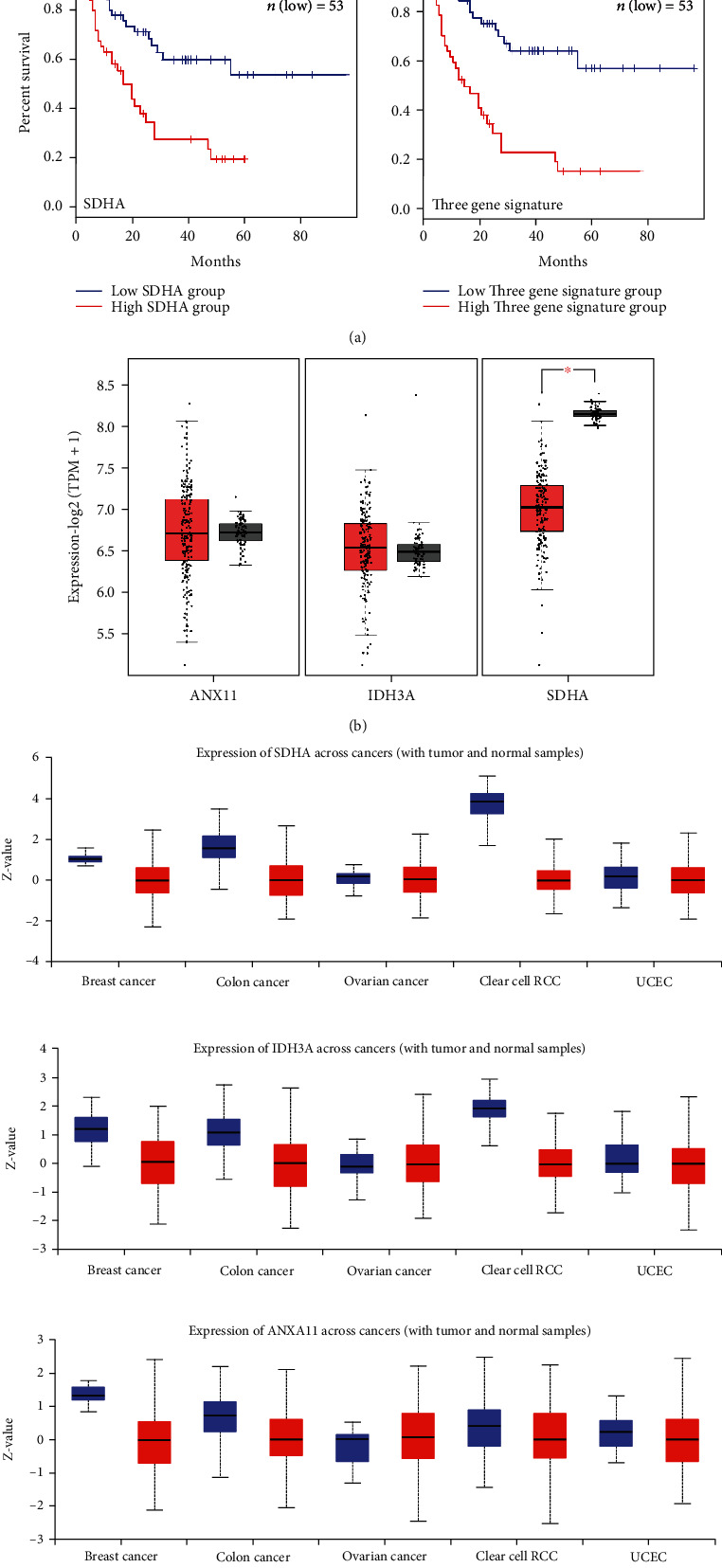
Identification of a three-gene signature correlated with prognosis in acute leukemia. (a) Kaplan-Meier survival analysis of indicated protein and signature using GEPIA web server and TCGA mRNA datasets on AML. (b) Protein levels of mRNA transcriptions of indicated proteins from AML patients were compared. Red: tumors; grey: normal tissues. (c) Abundances of indicated proteins in various cancer patients were compared using the UALCAN web server and CPTAC mass spectrometry datasets. Red: tumors; blue: normal tissues. (d) A model to summarize the major proteomic alterations required for cells to develop resistance toward death receptor-mediated apoptosis.

Given the central role of the mitochondria in regulating apoptosis, dysregulated mitochondrial functions may result in increased survival of tumors and poor prognosis. To further identify signaling nods critical for the survival of leukemia patients, we systematically performed the Kaplan-Meier survival analysis on most significantly upregulated proteins as indicated in Figure 3(c), using the GEPIA web server and TCGA datasets (see Materials and Methods for details). Due to data availability, TCGA data on AML, genetically related to ALL, was used instead. Although genetically distinguished, ALL and AML were similarly treated in clinical. Moreover, samples acquired from AML patients highly express DR5 and are sensitive to DR5-induced apoptosis [24, 25]. Therefore, the analysis on AML is likely to have a reference value for ALL. The Kaplan-Meier curve and log rank test analyses revealed that elevated mRNA levels of SDHA, IDH3A, and ANXA11 were individually associated with poor overall survival (OS) of AML patients (*P* < 0.05), while the three-gene signature consisting of all three genes (SDHA, IDH3A, and ANXA11) provided the highest predication power for OS of leukemia patients (*P* = 9.8E − 06) (Figure 4(a)). Notably, both the SDHA, an essential subunit of succinate dehydrogenase complex (SDC)/complex II, and the IDH3A, an isocitrate dehydrogenase catalyzing the oxidative decarboxylation of isocitrate, are mitochondrial proteins involved in regulating energy production [26–28]. On the other hand, annexin A11, or ANXA11, is a ubiquitously expressed member of the multigene family of Ca(2+)-regulated phospholipid-dependent and membrane-binding annexin proteins, acting to regulate a variety of cellular functions including proliferation, protein trafficking, and apoptosis [29]. These findings argued that dysregulated expression of SDHA, IDH3A, and ANXA11 might play an important role in determine cellular sensitivity apoptosis, thus serving as a potential vulnerability for therapeutic intervention with DR5-targeting agents. In supporting this notion, we showed that the expressions of these three genes have a much wider dynamic range in AML and several tumors compared to normal tissues, both at the level of mRNA and protein (Figures 4(b) and 4(c)).

The above findings were summarized in a mechanistic model where enhanced mitochondrial functions, especially elevated activities of energy metabolic regulators such as SDHA and IDH3A, and phospholipid-dependent signaling mediated by ANXA11, combined with decreased P53 signaling and ER stress response-related signaling, collectively contribute to the evasion of cell from death receptor-mediated apoptosis (Figure 4(d)).

## 4. Discussion

The ability of DR5 to selectively induce apoptosis in numerous cancers in vitro and in vivo ignited a series of enthusiasm to develop its agonists as therapeutics for AML and ALL. However, relevant clinical studies so far have been disappointing, partly due to poor understanding of underlining mechanisms regulating cellular sensitivity towards DR5-mediated cytotoxicity. To identify potential therapeutic vulnerability in ALL for DR5-targeted therapies, we first established a Jurkat derivative cell line that became resistant to DR5-agonistic antibody zaptuzumab. Jurkat is an in vitro cellular model for ALL. Subsequently, we systematically compared proteomes between wild-type and mutant cells by label-free quantitative mass spectrometry-based proteomic analysis, assisted by subcellular fractionation techniques, to identify proteomic changes necessary for cells to escape from DR5-induced apoptosis. Our analysis revealed an overall 17.4% qualitative difference between these two proteomes, demonstrating an extensive rewiring of signaling networks. Label-free quantification of subcellular fraction-specific proteomes and GO analysis further defined three main dysregulated protein clusters in drug-resisting Jurkat, potentially leading to enhanced respiration and energy production in the mitochondria and decreased transcription regulation and p53 signaling in the nucleus, consistent with the central role of the mitochondria in regulating apoptosis. Further, the Kaplan-Meier survival analysis using TCGA clinical mRNA databases on a group of most significantly changed proteins identified three proteins, SDHA, IDH3A, and ANXA11, correlated with poor prognosis of AML, closely related to ALL, when highly expressed. Moreover, a signature consisting all three genes significantly correlated with poor survival of AML patients when overexpressed.

Notably, two of the proteins, SDHA and IDH3A, key components of TCA, are involved in regulating energy metabolism in the mitochondria and considered to be actionable therapeutic targets in several cancers. For example, loss of SDHA enabling the utilization of glutamine as a fuel for the TCA cycle sensitizes AML cells towards venetoclax, a FDA-approved BCL-2 inhibitor [30], while overexpression of IDH3A, the third member of the isocitrate dehydrogenases (IDHs) family, has been associated with poor survival in breast cancer and liver cancer [31]. The third protein, ANXA11, is a mediator for Ca2+-dependent phospholipid signaling at the plasma membrane. Aberrant ANXA11 functions are involved in drug resistance and recurrence of systemic autoimmune disease and cancer [32]. While further investigations are required to establish clinical efficacy of these molecules, our work argues for the requirement of extensive metabolic reprogramming in the mitochondria, along with Ca^2+^-dependent phospholipid signaling at plasma membrane, for cells to develop resistance to DR5-mediated killing. Our results provided a unique insight on mechanistic vulnerabilities in leukemia cells, as well as opportunities to develop novel targeted strategies for leukemia.

## Data Availability

Data supporting the findings of this study are available from the authors upon request.

## Supplementary Materials

Supplementary Materials Figure S1: GO results for biological process on MS analysis of four subcellular fractions prepared from Jurkat cells. The results are representative of two independent biological replicates. Figure S2: comparison of the Venn diagram showing the overlaps of proteins identified in the indicated subcellular components in JurkatR (red) and Jurkat (blue). Figure S3: supplementary GO analysis of genes in Figure 3(c) for (a) biological process, (b) cellular components, and (c) molecular function. The color of the dot indicated number of genes enriched in corresponding pathways. Table S1: the number of identified proteins in subcellular compartments enriched from Jurkat and JurkatR.
