# Supplementary material for: Subcellular Proteome Analysis Reveals Apoptotic Vulnerability of T-Cell Acute Lymphoblastic Leukemia
Source: Biomed Res Int. 2022 Apr 15;2022:5504475. doi: 10.1155/2022/5504475 (PMC9033339; doi:10.1155/2022/5504475)

**Supplementary materials**

**Subcellular proteome analysis reveals apoptotic vulnerabilities of T-cell acute lymphoblastic leukemia**

Xiaolei Song^1#^, Xiaojing Wu^2#^, Zihan Zhang^2^, Zhangxiu Cui^2^,Yong Zheng^2^*, Jian Sun^1^*

*^1^Department of Molecular and Cellular Pharmacology, School of Pharmaceutical Science and Technology, Tianjin University, Tianjin, 300072, China; ^2^beijing*

*^2^ State Key Laboratory of Proteomics, Beijing Proteome Research Center, National Center for Protein Sciences (Beijing), Beijing Institute of Lifeomics, Beijing 102206, China*

# equal contribution

*To whom correspondence should be addressed:

Jian Sun (J. Sun)

Tel: 86-22-87401943; Fax: 86-22-27892025

E-mail: [sunj@](mailto:jsun@public3.bta.net.cn)tju.edu.cn (J. Sun)

Yong Zheng (Y. Zheng)

Tel: 86-010-61777070; Fax: 86-010-61777062

E-mail: zy@ncpsb.org.can

The supplementary material contains data for GO analysis for biological process (Fig. S1); Venn diagram for overlapping proteins (Fig. S2); GO analysis of genes in Fig. 3C (Fig. S3); and a table containing the total number of identified proteins (Tab. S1).

**Figure S1.** GO results for biological process on MS analysis of four subcellular fractions prepared from Jurkat cells. Results are representative of two independent biological replicates.

**Figure S2. Compare the** Venn diagram shows the overlaps of proteins identified in the indicated subcellular components in JurkatR (red) and Jurkat (blue).

**Figure S3.** Supplementary GO analysis of genes in Figure 3C for(a) biological process (b) cellular components and (c) molecular function. The color of the dot indicated number of genes enriched in corresponding pathways.

**Table S1.** The number of identified proteins in subcellular compartments enriched from Jurkat and JurkatR.

**Figure S1**


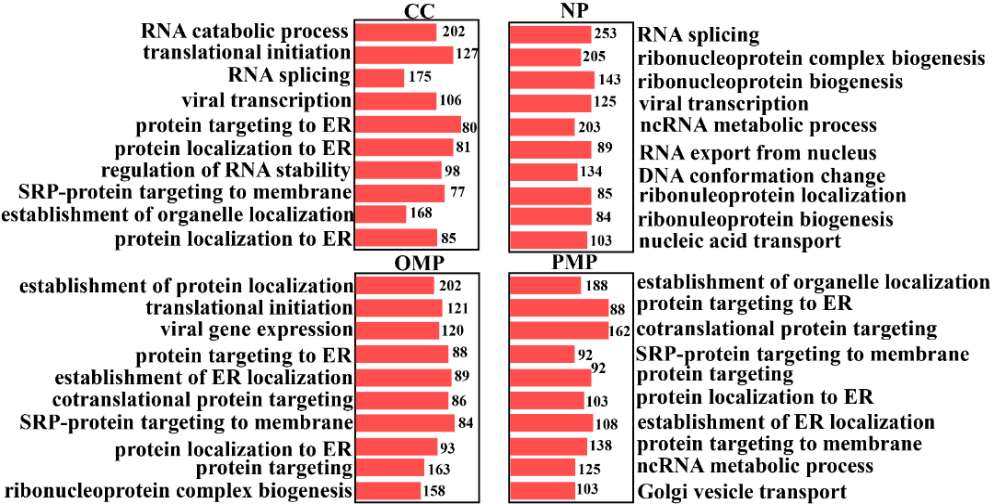


**Figure S2**


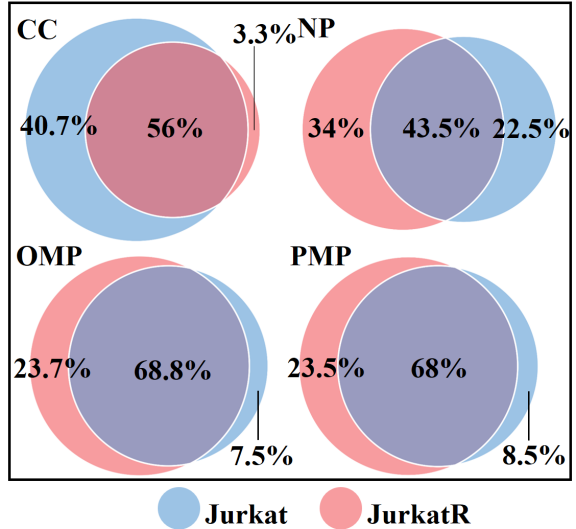


**Figure S3**


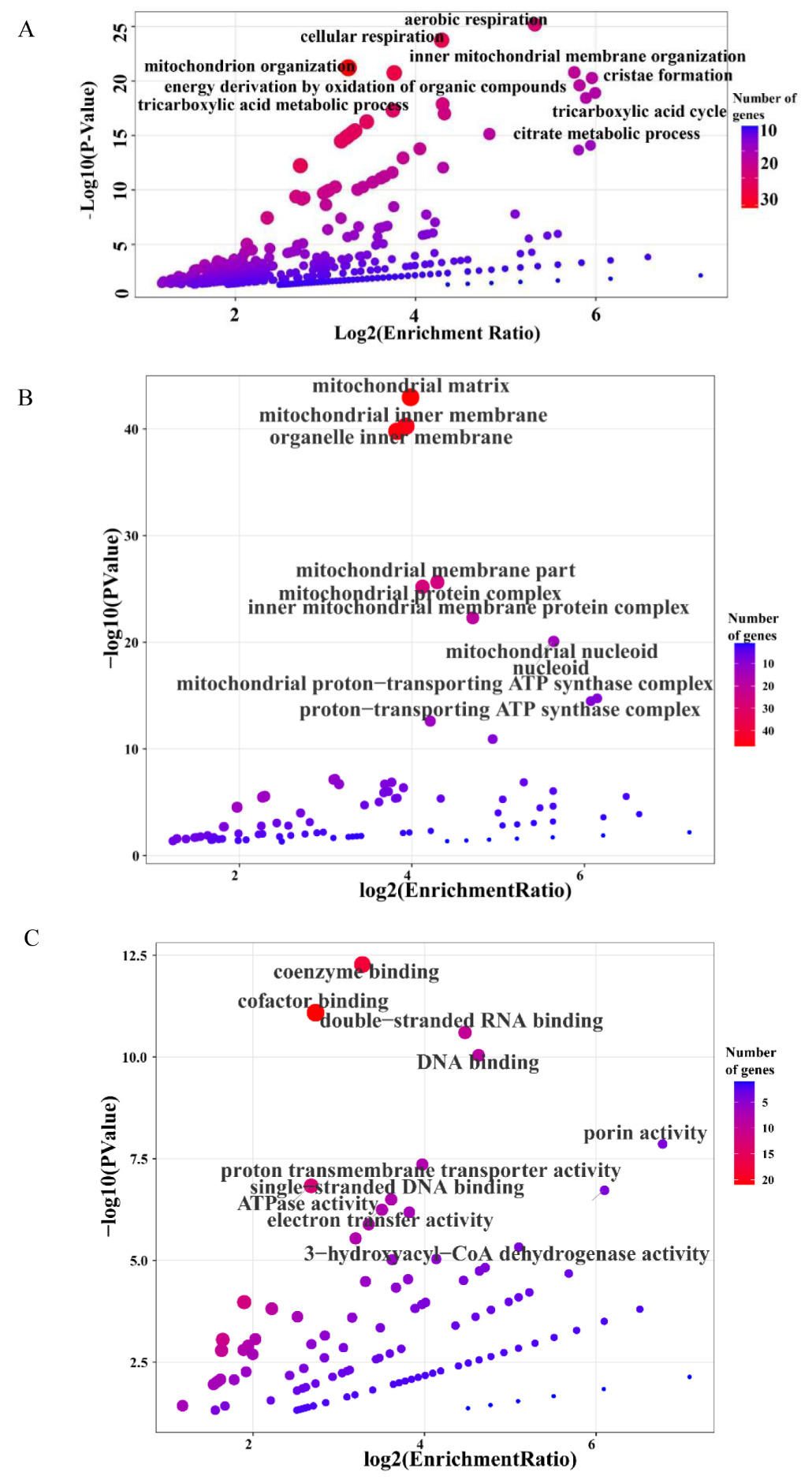


**Table S1**


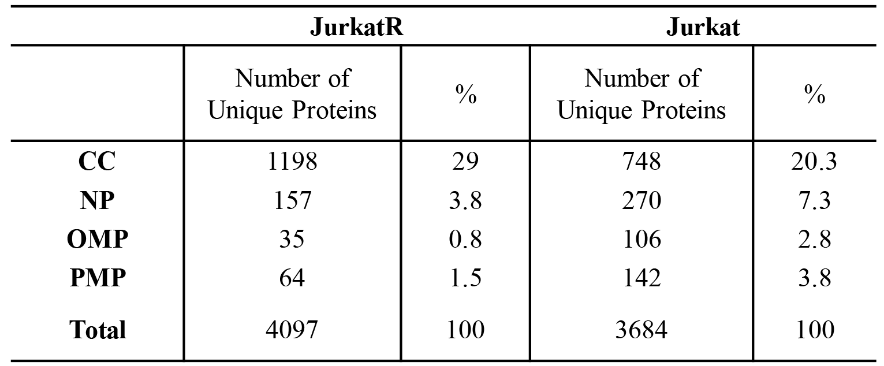

Supplement: Supplementary Materials — Figure S1: GO results for biological process on MS analysis of four subcellular fractions prepared from Jurkat cells. The results are representative of two independent biological replicates. Figure S2: comparison of the Venn diagram showing the overlaps of proteins identified in the indicated subcellular components in JurkatR (red) and Jurkat (blue). Figure S3: supplementary GO analysis of genes in Figure 3(c) for (a) biological process, (b) cellular components, and (c) molecular function. The color of the dot indicated number of genes enriched in corresponding pathways. Table S1: the number of identified proteins in subcellular compartments enriched from Jurkat and JurkatR. [file 5504475.f1.docx]
